# Seroprevalence of Equine Leptospirosis in the State of Goiás, Brazil

**DOI:** 10.3390/vetsci10100590

**Published:** 2023-09-25

**Authors:** Tatiana Nunes de Azevedo Romanowski, Ricardo Augusto Dias, Marcos Bryan Heinemann, Stephani Félix Carvalho, Tamires Ataides Silva, Andressa da Silva Martins, Geovanna Domingues da Cunha Caetano, Álvaro Ferreira Júnior, Jandra Pacheco dos Santos, Ana Carolina Borsanelli

**Affiliations:** 1 School of Veterinary Medicine and Animal Science, Universidade Federal de Goiás, Goiânia 74690-900, GO, Brazil; tatianaromanowski@hotmail.com (T.N.d.A.R.); mv.stephanicarvalho@gmail.com (S.F.C.); tamires_ataides@discente.ufg.br (T.A.S.); zandressa@discente.ufg.br (A.d.S.M.); geovanna_domingues@discente.ufg.br (G.D.d.C.C.); alvaro.ferreira@ufg.br (Á.F.J.); 2 School of Veterinary Medicine, University of São Paulo, São Paulo 05508-270, SP, Brazil; ricardodias@usp.br (R.A.D.); marcosbryan@usp.br (M.B.H.); 3 Unigoiás, University Center of Goiás, Goiânia 74423-115, GO, Brazil; jandra.santos@yahoo.com.br

**Keywords:** *Leptospira* spp., zoonotic diseases, microscopic agglutination test, seroepidemiology, equine health

## Abstract

**Simple Summary:**

Leptospirosis is a zoonosis of bacterial origin caused by spirochetes of the genus *Leptospira*. It is distributed worldwide and has a great impact on public health and the agricultural economy. In Latin America, leptospirosis is a neglected tropical disease, despite being infectious, zoonotic, with an acute course, and eventually lethal. In the Brazilian state of Goiás, which is located in the tropical savanna (Cerrado), data on the seroprevalence of equine leptospirosis are scarce and restricted to a few municipalities. Thus, the present study aimed to evaluate the seroprevalence of *Leptospira* spp. in 294 equine herds in the state of Goiás, Brazil. The results shed light on the significant and widespread prevalence of *Leptospira* in both farms (72.9%) and horses (61.6%) in Goiás State. These results were indicative of a complex epidemiological landscape characterized by the predominance of serogroups such as Djasiman, Icterohaemorrhagiae, and Australis. Therefore, our findings underscore the critical significance of implementing sanitation measures within the environment and conducting health education initiatives to effectively prevent and control equine leptospirosis within the state, with particular emphasis on the necessity of vaccination.

**Abstract:**

Leptospirosis, caused by Leptospira, is a zoonotic disease that, in horses, is linked to abortions, uveitis, and sporadic occurrences of liver and kidney disease, often resulting in significant economic losses for farmers. Research on the prevalence of leptospirosis in horses in the central-west region of Brazil has been relatively scarce. Thus, the present study aimed to determine the prevalence of leptospirosis in equine herds in the state of Goiás (Central Brazil). Blood samples were collected from 894 equids at 294 randomly selected farms divided into three different strata according to their herd characteristics. The microscopic agglutination test for the detection of anti-*Leptospira* agglutinins was carried out and the results showed that among the 294 sampled farms, 213 (72.9%; CI 95% 71.7–78.9) had one or more animals positive for leptospirosis, and of the 894 horses sampled, 513 (61.6%; CI 95% 54.3–69.0) were seropositive for leptospirosis. Djasiman, Icterohaemorrhagiae, and Australis were the most prevalent serogroups. The results showed a high prevalence of seropositive animals and a widespread distribution of positive farms in the state of Goiás. Thus, environmental sanitation measures and health education to prevent and control equine leptospirosis in the state are required.

## 1. Introduction

*Leptospira* spp. are Gram-negative spirochetes that cause leptospirosis and can infect various mammalian host species, including horses and humans. Bacteria can be transmitted indirectly through exposure to polluted water or soil, or directly through contact with animal urine [1]. Among domestic and wild animals, leptospires can persist in chronic renal carriers and subsequently be expelled through urine, thus acting as potential reservoirs of infection for humans and other animals [2].

Leptospirosis is considered the most prevalent zoonotic disease globally, with approximately 1.03 million clinical cases occurring annually in humans, leading to 58,900 deaths. This disease also ranks as a primary cause of illness among various species of domestic animals [3]. However, in Latin America, leptospirosis remains as a neglected tropical disease, despite its infectious, zoonotic nature, acute course, and potential lethality [4]. 

In horses, the clinical manifestations of leptospirosis can vary depending on the severity of the disease. In mild cases, affected animals may exhibit clinical signs such as fever, lethargy, and loss of appetite. In more severe forms of the illness, noticeable clinical signs may include jaundice, anemia, the development of petechial hemorrhages, and even renal failure [5]. Furthermore, when pregnant mares contract the infection, it can lead to placentitis and late-term abortion without any preceding clinical indicators [6]. Notably, uveitis represents a significant complication arising from systemic infection in horses [7]. However, subclinical forms are the most prevalent, especially in regions where the disease is endemic [1].

Despite its significance in horses, leptospirosis, which is an important cause of abortions, the births of weak or premature animals, eye issues, and interstitial nephritis-related deaths, has received limited attention until recently [8]. Nevertheless, the recognition of the significant economic consequences associated with leptospiral infections, such as the loss of valuable foals or career-ending recurrent uveitis in horses, has led to changes in attitudes.

Leptospirosis in horses not only causes economic losses, but also poses a risk of human infection, particularly in occupational settings where individuals have prolonged contact with infected horses [9]. In addition, evidence of the presence of *Leptospira* in the urine of seropositive horses raised near seropositive households suggests the potential of these animals as sources of environmental contamination [10]. Thus, veterinarians, waste recyclers, and dairy farm employees stand out among the most exposed professionals, to both seropositive horses and environments contaminated with *L. interrogans* [9].

Studies on seroprevalence and isolation have shown that horses are susceptible to a wide range of incidental infections, including serovars of the Pomona and Grippotyphosa serogroups, as well as the Icterohaemorrhagiae, Autumnalis, Sejroe, Canicola, and Ballum serogroups [2]. However, few studies have evaluated the prevalence of leptospirosis in horses in the Central-West region of Brazil and in the state of Goiás, located in the tropical savanna (Cerrado), data on the seroprevalence of equine leptospirosis are scarce and restricted to a few municipalities [11,12]. Thus, the aim of the present study was to determine the seroprevalence of *Leptospira* spp. in equine herds in the state of Goiás, Brazil.

## 2. Materials and Methods

### 2.1. Study Area and Animals

The state of Goiás is in the Central-West region of Brazil, with an area of 340,243 km^2^, equivalent to approximately 4% of the Brazilian territory. A tropical savanna (Cerrado) climate is dominant, with rainy summers and dry winters. It has a high temperature range (16 °C to 34 °C), with an average of 26 °C; the amount of rainfall varies from 1500 mm to 1800 mm/year. The average rainfall in the period from November to January is 285 mm, humidity of 79%, average minimum temperature of 23 °C and average maximum temperature of 33 °C. In 2021, the state’s equine herd included 393,676 animals distributed across 91,880 farms, representing 7.3% of the national herd [13].

### 2.2. Sample Collection

In order to assess the seroprevalence of herds and identify equines that tested positive for leptospirosis, a two-stage sampling approach was implemented. The initial stage involved the selection of farms, followed by the subsequent selection of individual equines within those chosen farms [14]. To improve the sampling universe, herds in Goiás were divided into three strata according to the type of horse breeding. Stratum 1: farms with horses only; Stratum 2: farms with horses and cattle; Stratum 3: farms in urban areas as described by Pádua et al. [14]. The collection of clinical samples used in this study were conducted as part of an epidemiological survey for equine infectious anemia in the state using specific parameters for this disease [14]. Thus, the selection of farms was conducted in a random manner using the Agrodefesa database of registered equine farms. The determination of the number of animals to be included in the sample was made with the aim of achieving a minimum of 90% specificity at the herd level. The sample size was calculated with the assumption of herds consisting of 5–10 horses, given that the average herd size within each stratum was roughly five animals. Consequently, for farms having fewer than nine equids, blood serum was acquired from every single animal. Conversely, for farms with herds comprising ten or more animals, samples were collected from a total of nine animals [14]. Blood was collected by 27 field teams from the local veterinary service, Goiás Agricultural Defense Agency (Agrodefesa) between November 2020 and January 2021. Blood samples were collected aseptically by jugular venous puncture, centrifuged to obtain serum, identified, and stored in duplicate in 1.5 mL microtubes at −20 °C until processing. This study was approved by the Ethical Committee of Animal Use of the Federal University of Goiás (protocol No. 108/22).

### 2.3. Laboratory Analysis

The microscopic agglutination test (MAT) for the detection of anti-*Leptospira* agglutinins [15] was performed at the Laboratory of Leptospirosis Diagnosis of the School of Veterinary and Animal Science of the Universidade Federal de Goiás (EVZ/UFG). A panel of 22 *Leptospira* serovars representing 13 serogroups was used (*Leptospira interrogans* serovars: Australis, Bratislava, Autumnalis, Canicola, Djasiman, Sentot, Hebdomadis, Copenhageni, Icterohaemorrhagiae, Pomona, Pyrogenes, Hardjo-prajitno, Hardjo (CTG strain), Wolffi; *Leptospira borgpetersenii* serovars: Castellonis, Hardjobovis, Tarassovi; *Leptospira kirschneri* serovars: Butembo, Grippotyphosa; *Leptospira santarosai* serovar: Shermani; and *Leptospira biflexa* serovar: Patoc, Andamana). Suspensions of live bacteria grown for seven days in EMJH medium were used. The cut-off point adopted for MAT was a titer of 100 [16]. The sera of reactive animals were tested at maximum dilution of 1:800. Dilutions with 50% or more agglutination under dark-field microscopy were considered reagents. The serovar with the highest titer was used to characterize the most likely serovar.

### 2.4. Statistical Analysis

To estimate the prevalence of *Leptospira* infection, the relative weights for both the chosen farms and the tested animals within those farms were analyzed. These weighted values were subsequently calculated to derive prevalence estimates in the state. Confidence intervals were obtained using the binomial logistic regression model. These analyses were conducted utilizing the R software [17].

## 3. Results

### 3.1. Seroprevalence

**Figure 1:**
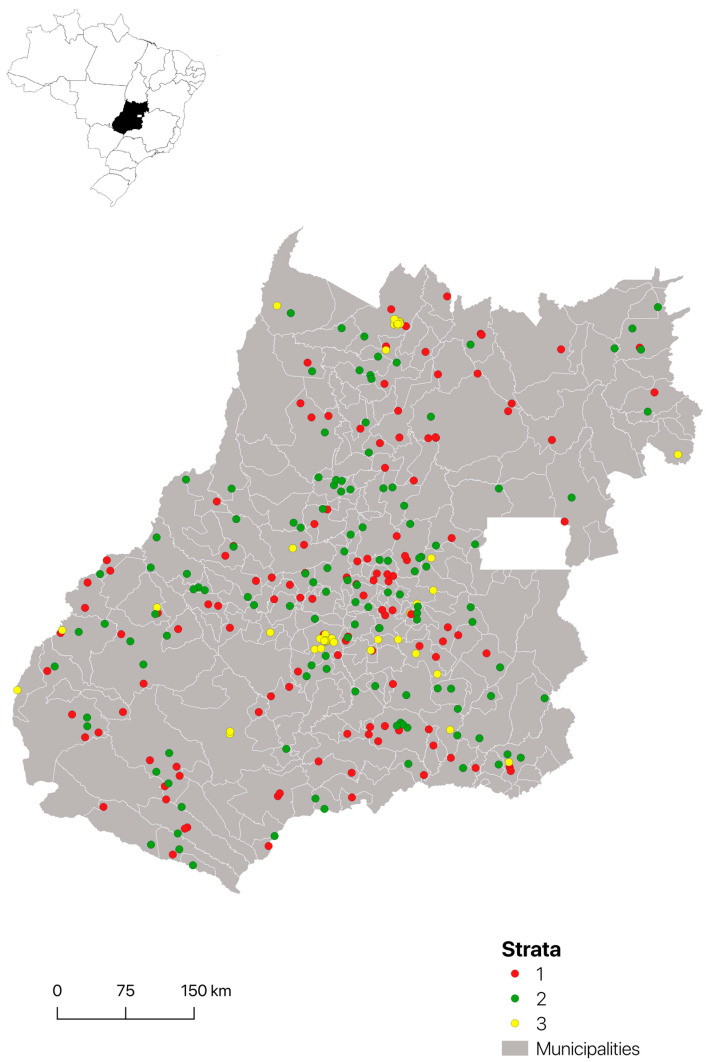
Geographical location of the surveyed farms of the State of Goiás according to the stratum.

Among the 294 sampled farms, 122 (41.5%) belonged to Stratum 1, 123 (41.8%) to Stratum 2, and 49 (16.7%) to Stratum 3 (Figure 1). Serum samples were collected from 894 animals, including 787 (88%) horses, seven (0.78%) donkeys, and 100 (11.2%) mules. Of these, 390 animals belonged to Stratum 1 (farms with horses only), 380 to Stratum 2 (farms with horses and cattle), and 124 to Stratum 3 (farms in urban areas). The average number of animals sampled per farm was 2.27, ranging from 1 to 9 animals per farm. 

**Figure 2:**
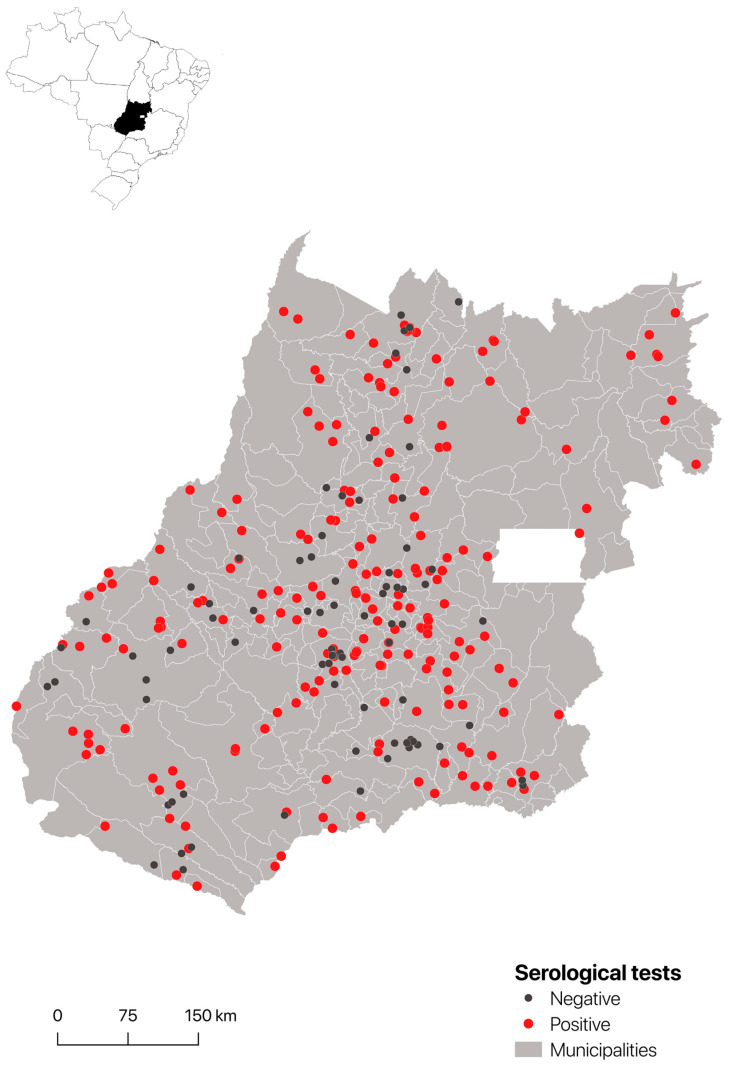
Geographical location of the surveyed farms of the State of Goiás and the positives and negatives farms to *Leptospira* infection.

The seroprevalence per farm and animal is described in Table 1 and Table 2 and illustrated in Figure 2. The apparent seroprevalence per animal was 61.6% (513/894, CI 95% 54.3–69.0) and the seroprevalence per farm was 72.9% (213/294, CI 95% 71.7–78.9). Farms with horses and cattle (Stratum 2) showed the higher seroprevalence of focus (73.7%) and the prevalence of seropositive horses was higher in farms with horses only (Stratum 1; 65.1%). However, among the strata evaluated, there were no significant differences in the number of seropositive animals.

### 3.2. Serology

Among the 894 serum samples evaluated, 513 (61.6%) tested positive for anti-*Leptospira* antibodies. At least one animal tested positive for each of the 22 serovars tested. The highest frequencies were found in serogroups Djasiman (Djasiman: 16.4%; Sentot: 5.7%), Icterohaemorrhagiae (Icterohaemorrhagiae: 12.1%; Copenhageni: 1.2%), Bratislava (Bratislava: 5.5%; Australis: 1.4%), and Pomona (Pomona: 5.5%; Pomona GR6: 0.6%) (Table 3). Titers ranging from 100 to 800 were identified for all the serovars. Icterohaemorrhagiae was the most prevalent serovar in horses from stratum 3 (farms in urban areas) and Djasiman was the most prevalent serovar in horses from strata 1 (farms with horses only) and 2 (farms with horses and cattle).

## 4. Discussion

Leptospirosis is a zoonotic bacterial infection caused by Gram-negative spirochetes belonging to the genus *Leptospira*. In Brazil, leptospirosis is prevalent among domesticated animals and has been documented in swine, sheep, goats, and bovines [18,19,20,21], with seroprevalence rates ranging from 20% to 50%. The examination of seroprevalence in horses is important due to their considerable economic importance. Similar to humans, accidental infections are most prevalent in tropical and humid regions where poor sanitation, inadequate rodent control, and a combination of domestic animal management practices creates an environment conducive to the contamination of various strains of *Leptospira*. These conditions also facilitate the prolonged survival of these strains in the environment [1].

Leptospirosis in horses is typically suspected when there are frequent ocular issues, although it is important to note that many cases remain asymptomatic. The confirmation of diagnosis can be achieved through either culturing the bacteria or conducting serological tests. Antibody detection using the microscopic agglutination test (MAT) is considered a reference test by the World Health Organization for the diagnosis of leptospirosis in humans and animals [22]. This technique has high specificity and sensitivity and uses representative strains of known serovars in the specific country or region [1]. This method frequently permits the identification of the suspected causative serogroup, offering valuable epidemiological data about the prevalence of circulating serogroups [22]. However, it is a technique that requires serovars to be cultured, and this maintenance is relatively complex, as live cultures demand constant care and ongoing maintenance. The results of the present study revealed that 72.95% of the evaluated farms in the state of Goiás had at least one seropositive equine for leptospirosis and that of the 894 sera evaluated by the microscopic agglutination test, 513 (61.6%) presented positive reactions, with titers equal to or greater than 1:100.

The prevalence of anti-*Leptospira* antibodies in horses may vary according to the geographic region and the serovars evaluated. In Brazil, the first report of leptospiral infections in horses was registered in army horses in São Paulo with a prevalence of 16.9% seropositive animals [23]. The seroprevalence of anti-*Leptospira* antibodies in horses ranges from 4.5% to 90.7%, with an average of 45%, depending on the geographic region and serovar [2]. In several studies, it was reported that in the Southeast region the prevalence is approximately 17.9% to 71.9% [24], in the South region, approximately 45.9% [16], and between 23% and 100% in the North [25] and Northeast [26] regions. Among the most prevalent serogroups, Icterohaemorrhagiae was the most frequent (47.8%), followed by Australis [2].

In previous studies conducted in the Central-West region, the prevalence of seropositive horses ranged from 9.3% to 36.2% [26]. However, only two studies on equine leptospirosis have been performed in Goiás, and these were restricted to a few municipalities. In these studies, the frequency of the disease in horses and the prevalence of seropositive animals ranged from 14.4% [11] to 45.05% [12]. Thus, the results of the present study indicate a higher prevalence of seropositive animals than in previous studies, and this is the first study in which animals from the entire state were evaluated. 

Regarding the geographical distribution of equine leptospirosis in Goiás, it was observed that in addition to the high prevalence, *Leptospira* infection was distributed across the state (Figure 2). These results show that equine leptospirosis is an endemic disease in equine herds in the state of Goiás, highlighting the need for prevention and control measures related to this disease, including horse vaccination. 

Farms with horses and cattle (Stratum 2) showed the higher seroprevalence of focus (73.7%) and prevalence of seropositive horses was higher in Stratum 1 (65.1%). However, among the strata evaluated, there was no significant difference in the number of seropositive animals. Regarding the evaluated serovars, it was observed that Djasiman and Sentot predominated in farms with horses (Stratum 1) and farms with horses and cattle (Stratum 2). This can be attributed to the fact that in rural areas, animals have greater contact with wildlife, such as the maned wolf (*Chrysocyon brachyurus*), coati (*Nasua nasua*), crab-eating fox (*Cerdocyon thous*), crab-eating raccon (*Procyon cancrivorus*), hoary fox (*Lycalopex vetulus*), and nine-banded armadillo (*Dasypus novemcinctus*). Djasiman was the most common serogroup identified in these wild species by Fornazari et al. [27]. On the other hand, the Icterohaemorrhagiae serovar was more prevalent on farms located in urban areas (Stratum 3), likely due to the increased contact of animals raised in urban environments with synanthropic rodents. The Australis serovar showed a similar prevalence in all three strata, as expected, since equids are the primary reservoirs of this serovar. These results highlight the complexity of the epidemiological chain of leptospirosis.

In Brazil, Icterohaemorrhagiae, Australis, and Pomona are serogroups commonly associated with equine leptospirosis [2]. In the present study, 22 serovars representing 13 serogroups were used in the MAT and at least one positive animal tested positive for each serovar tested. The predominant serogroups identified were Djasiman, Icterohaemorrhagiae, Bratislava, and Pomona. The Djasiman serogroup was the most prevalent, with emphasis on the serovars Djasiman and Sentot which were identified in 84 (16.04%) and 29 (5.7%) horses, respectively. The serovar Djasiman was first reported in Indonesia, with initial assumptions suggesting its confinement to the South-East Asian region [28]. However, this serovar was recently identified in horses in South Africa [29] and it was most prevalent in dairy cattle from Southern Brazil [30]. In addition, a high frequency of seroreactivity for Djasiman serogroups was reported in several wild animal species from the Pantanal biome, particularly the pampa deer (*Ozotoceros bezoarticus*) [27,31]. This serovar has also been associated with human leptospirosis [32,33]; however further studies on its zoonotic characteristics are necessary. This is the first report of this serovar in horses in Brazil.

Positive results for serovar Sentot suggested that the sampled equids had contact with environments where wild animals were present, as these are considered responsible for maintaining this agent in nature [34]. The first identification of this serovar in domestic animals in Brazil was reported by Herrmann et al. [35] who tested sheep in Rio Grande do Sul. Sentot was among the most prevalent serovars identified in horses in the Amazon region [36]; however, few studies related to the seroprevalence of leptospirosis in horses have evaluated the presence of this serovar. Thus, in the present study, the high prevalence of these two serovars can indicate a large exposure of the sampled equids to wild species.

The Icterohaemorrhagiae serogroup also showed a high prevalence in the sampled animals, which could indicate direct or indirect contact of horses with synanthropic rodents, since leptospires are maintained in the environment by the contaminated urine of these animals which are renal carriers [37,38]. The Icterohaemorrhagiae serovar was the most prevalent in this serogroup, identified in 62 (12.1%) horses. This serovar is highly prevalent in horses from the states of São Paulo [39], Minas Gerais [40], Paraná [41], Rio Grande do Sul [42], and Rio de Janeiro [43]. This serovar was most prevalent in horses from the microregion of Goiânia in the previous study carried out in Goiás [12]. In horses, infections resulting from incidental serogroups such as Icterohaemorrhagiae typically result in acute systemic disease [8]. However, in the present study, the clinical parameters of the horses were not assessed. 

Icterohaemorrhagiae serovar identification is extremely relevant because it is responsible for the vast majority of *Leptospira* infections in humans [44,45]. Hamond et al. [18] identified the presence of leptospiral DNA in the urine of seropositive horses, indicating that these animals can act as carriers of this microorganism, which deserves attention from a public health perspective. However, additional studies are required to elucidate the importance of naturally infected horses in the epidemiological chain of lepstopirosis.

Serovar Copenhageni, a member of the Icterohaemorrhagiae serogroup, was identified in only six (1.2%) horses. The rat (*Rattus novergicus*) is the natural host of this serovar [46] and represents an important etiological agent of leptospirosis, both in asymptomatic cases and in cases of reproductive disorders in females [47]. Copenhageni was the most frequent serovar found in Thoroughbred horses from Rio de Janeiro, Brazil. After a severe storm, the premises of the animals remained flooded for 72 h [18], and the horses were probably exposed to rat urine.

Among the serogroups found in this study, Australis showed a high prevalence in the horses sampled, with an emphasis on the Bratislava serovar (5.5%). The high prevalence of this serovar was expected because horses act as a reservoir species, and it has been identified in studies on horses in some regions of Brazil [16,43] and worldwide [48,49]. Bratislava is associated with subclinical infections in horses [1] and has recently been associated with active ocular disease, blindness, and poor prognosis in horses with uveitis [7]. The significance of Bratislava in the etiology of leptospirosis in horses has gained increasing attention. The infection mainly leads to reproductive issues with no substantial biochemical or hematological changes, suggesting a persistent, asymptomatic, and localized infection in horses.

In this study, the Pomona serogroup was identified in 28 horses (6.1%). In a previous study conducted in a micro-region of the state of Goiás, Pomona was the second most prevalent serovar and was identified in 13.4% of the 182 horses evaluated [12]. This serogroup is considered the most adapted to swine species [50], and the results of the present study may indicate the contact of horses with these animals on farms. The Grippotyphosa serogroup, commonly associated with ophthalmic alterations in horses [1], was identified in only eight animals (1.6%), and the serovar Canicola, which has dogs as its main host [1], was not relevant in terms of the number of reactive animals.

Regarding the factors associated with equine leptospirosis, working horses tend to have a higher prevalence of anti-*Leptospira* antibodies than sport animals [51]. In the present study, the horses were classified according to their farm profiles and the use of animals. However, no significant difference was observed in the prevalence of seropositive animals on farms with horses only (Stratum 1), farms with horses and cattle (Stratum 2), farms in urban areas (Stratum 3), or in the function of the animals. 

Controlling leptospirosis presents significant challenges, mainly due to variations in control methods based on the host species and specific serogroups of leptospires involved. Knowledge of the prevalent serogroups for each host is crucial [52], as control approaches differ depending on the infecting strain. However, few studies have evaluated the epidemiological status of this disease in domestic animals, especially in tropical environments. This lack of information hampers the effectiveness of the control programs.

Typically, control strategies involve antibiotic therapy for carriers, implementation of appropriate handling and environmental measures, and most importantly, systematic immunization of susceptible animals [8]. Therefore, vaccines must be effective against the most prevalent leptospiral serovars in a species or region. Environmental measures also vary depending on the infecting strain, making accurate diagnosis crucial for a successful control program [53].

Livestock vaccination is instrumental in reducing urinary shedding and the risk to human handlers, especially when combined with appropriate education programs, community awareness, and hygiene practices. Support from authorities responsible for human and veterinary public health administration is crucial [6]. Successful vaccination programs require continuous epidemiological studies to monitor the incidence of various *Leptospira* serovars in the population. The complexity of leptospirosis epidemiology, coupled with the diversity of wildlife and challenges in diagnosing silent reproductive diseases, presents significant hurdles for leptospirosis control. Nevertheless, to mitigate the impact of clinical diseases in horses, a triad of approaches comprising antibiotic therapy, vaccination, and environmental management are essential [52].

## 5. Conclusions

In conclusion, this study demonstrated the high and widespread prevalence of *Leptospira* on farms and in horses in Goiás State, with a predominance of serogroups Djasiman, Icterohaemorrhagiae, and Australis, suggesting a complex epidemiology. Thus, our results reinforce the importance of environmental sanitation measures and health education for the prevention and control of equine leptospirosis within the state, particularly regarding the need for vaccination. 

## Figures and Tables

**Table 1:** Apparent seroprevalence of anti-*Leptospira* antibodies among farms with a focus of equine leptospirosis in three strata in the State of Goiás, 2021.

Stratum	Farms			
	N	Focus *	%	CI_95%_
1	122	85	68.3	59.6–76.2
2	123	94	73.7	65.3–80.9
3	49	34	67.7	52.9–79.9
Total	294	213	72.9	71.7–78.9

* Farms with at least one animal seropositive for lepstospirosis. Stratum 1: farms with horses only; Stratum 2: farms with horses and cattle; and Stratum 3: farms in urban areas.

**Table 2:** Apparent seroprevalence of anti-*Leptospira* antibodies in sampled equids in three strata in the State of Goiás, 2021.

Stratum	Animals			
	N	MAT (+)	%	CI_95%_
1	390	207	65.1	50.0–78.4
2	380	233	61.2	53.1–68.9
3	124	73	58.5	49.7–67.0
Total	894	513	61.6	54.3–69.0

Stratum 1: farms with horses only; Stratum 2: farms with horses and cattle; and Stratum 3: farms in urban areas.

**Table 3:** Frequency of the most probable serogroups and serovars by stratum and total of seropositive equids.

Serogroup	Serovar	Stratum 1	Stratum 2	Stratum 3	Goiás (n/%)
Djasiman	Djasiman	37 (18.0)	39 (17.0)	8 (10.4)	84 (16.4)
	Sentot	14 (6.8)	13 (5.7)	2 (2.6)	29 (5.7)
Icterohaemorrhagiae	Icterohaemorrhagiae	26 (12.7)	15 (6.5)	21 (27.3)	62 (12.1)
	Copenhageni	3 (1.5)	2 (0.9)	1 (1.3)	6 (1.2)
Australis	Bratislava	10 (4.9)	15 (6.5)	3 (3.9)	28 (5.5)
	Australis	3 (1.5)	3 (1.3)	1 (1.3)	7 (1.4)
Pomona	Pomona	14 (6.8)	13 (5.7)	1 (1.3)	28 (5.5)
	Pomona GR6	1 (0.5)	2 (0.9)	0	3 (0.6)
Ballum	Castellonis	8 (3.9)	9 (3.9)	3 (3.9)	20 (3.9)
Sjeroe	Hardjobovis	7 (3.4)	3 (1.3)	2 (2.6)	12 (2.3)
	Hardjo (CTG)	0	1 (0.4)	1 (1.3)	2 (0.4)
	Hardjo-prajitno	0	1 (0.4)	0	1 (0.2)
	Wolffi	1 (0.5)	0	0	1 (0.2)
Autumnalis	Autumnalis	3 (1.5)	6 (2.6)	0	9 (1.8)
	Butembo	0	0	1 (1.3)	1 (0.2)
Grypphotyphosa	Grypphotyphosa	2 (1.0)	6 (2.6)	0	8 (1.6)
Shermani	Tarassovi	0	2 (0.9)	0	2 (0.4)
Andamana	Andamana	1 (0.5)	0	0	1 (0.2)
Seramanga	Patoc	22 (10.7)	37 (16.2)	12 (15.6)	71 (13.9)
	Without more prevalent serovar *	53 (25.8)	62 (27.1)	21 (27.2)	136 (26.5)
Total		205 (100)	229 (100)	77 (100)	513 (100)

* Tie of the biggest titers. Stratum 1: farms with horses only; Stratum 2: farms with horses and cattle and Stratum 3: farms in urban areas.

## Data Availability

The data are available from the corresponding author upon reasonable request.
